# Branched Pd@Rh core@shell nanocrystals with exposed Rh {100} facets: an effective electrocatalyst for hydrazine electro-oxidation

**DOI:** 10.1038/s41598-017-16776-6

**Published:** 2017-11-28

**Authors:** Guojing Wang, Shengchang Jing, Yiwei Tan

**Affiliations:** State Key Laboratory of Materials-Oriented Chemical Engineering, School of Chemistry and Chemical Engineering, Nanjing Tech University, Nanjing, 210009 China

## Abstract

Shape control of noble metal (NM) nanocrystals (NCs) is of great importance for improving their electrocatalytic performance. In this report, branched Pd@Rh core@shell NCs that have right square prism-like arms with preferential exposure of Rh {100} facets (denoted as b-Pd@Rh-NCs thereafter) are synthesized and utilized as an electrocatalyst for the hydrazine electrooxidation (HEO) in acidic and alkaline electrolytes. The b-Pd@Rh-NCs are obtained by the heteroepitaxial growth of Rh on the pre-formed branched Pd NCs (denoted as b-Pd-NCs thereafter) core in the presence of poly(vinyl pyrrolidone) (PVP) and bromide ions. A comparative analysis of the voltammetric data for the HEO shows a higher activity on the b-Pd@Rh-NCs exposed with Rh {100} faces than on Rh black, the b-Pd-NCs, and Pd black in acid and alkaline solutions, indicating a structure sensitivity of the reaction. Analysis of the products from the b-Pd@Rh-NCs catalysed HEO reveals a very high hydrazine fuel efficiency, as determined by on-line differential electrochemical mass spectrometry (DEMS).

## Introduction

Increased risk in climate changes and the consequent demands on clean energy conversions compel and drive the development of fuel cell technologies as a viable scheme to replace fossil-fuel based energy sources. Among various fuel cells, hydrazine-air fuel cells gain more attention because hydrazine can produce a very high energy release per unit volume after oxidation with O_2_ (the reaction enthalpy of −19.7 × 10^3^ kJ L^−1^ for the reaction N_2_H_4_ (l) + O_2_ (g) = N_2_ (g) + 2H_2_O (l)) and, moreover, gives zero emission (nitrogen and water are the only products of hydrazine oxidation)^1–14^. Furthermore, the fuel cells powered by hydrazine have a very high theoretical cell voltage (1.56 V) and thus enable higher volumetric energy and power densities than hydrogen−oxygen fuel cells. HEO undergoes a multi-step successive electrochemical dehydrogenation process and is highly dependent on the surface crystallographic structure of metallic electrodes^15,16^. For example, bulk Pt(100) and Rh(100) surfaces show a higher electrocatalytic activity than the corresponding (111) and (110) surfaces in perchloric acid solution^15^. Rosca *et al*. demonstrated a different activity trend for bulk Pt electrode (i.e., Pt(110) > Pt(100) > Pt(111)) in alkaline electrolyte^16^. Hence, the exploration of electrocatalysts with a suitable surface structure is of imperative significance for fundamentally understanding and optimizing the electrocatalytic process of hydrazine fuel cells.

Compared to bulk single-crystal electrocatalysts, nanostructured counterparts with well-defined exposed surfaces could show a higher mass activity and a lower onset potential due to a dramatic increase in exposed active sites, thus enabling a better performance while requiring a much less loading level of catalyst. The composition, morphology, and surface structures of NM NCs are the basic motifs of electrocatalyst design. As such, fabrication of NM NCs with unique morphology and well-defined surface structures is highly desirable for improving electrocatalytic activity. Up to now, a variety of NM NCs, such as Au^8,13,17,18^, Pd^7,19,20^, Pt^14,18,21,22^, and Ag^23^, have been used as electrocatalysts for HEO. However, to our knowledge, there have been no reports to date of rhodium-based nano-electrocatalysts with well-defined surface structures and their electrocatalytic activity towards HEO.

Although much effort was devoted to prepare shape-controlled zero-dimensional Rh polyhedral NCs with well-defined crystal facets to fulfill the requirement of various catalysis applications^24–33^, one dimensional NCs containing Rh component and selectively exposing well-defined facets, which represent an important class of catalysts due to access to long-range, large area facets, have not been reported thus far. At the same time, core-shell nanocatalysts are fascinating because of their unique properties, such as favorable synergistic electronic effects and unusual crystallographic structure^34,35^. Herein, we successfully synthesized b-Pd@Rh-NCs with exposed Rh {100} facets on their surface using the pre-synthesized b-Pd-NCs as the core template. In contrast to those previous branched NM core−Rh shell NCs with ambiguous surface structures^27,36–38^, our b-Pd@Rh-NCs spur new opportunities for catalytic applications due to the favorable surface atom arrangement. The preceding surface structure-dependent catalytic activity for HEO renews an impetus to evaluate the structure-activity relationship for the b-Pd@Rh-NCs in this context. It was found that the b-Pd@Rh-NCs show a higher activity towards the HEO than Rh black, the b-Pd-NCs, Pd black, and the bulk Rh (100) single crystal by comparing the HEO on these electrocatalysts in acid and alkaline media. Considering that DEMS technique is an effective approach to detecting different volatile products during electrode reaction and assessing cell fuel efficiency^39^, we further demonstrate a mechanistic scheme of the HEO on the b-Pd@Rh-NCs by combining voltammetry and DEMS.

## Results and Discussion

### Synthesis and characterization of b-Pd@Rh-NCs

**Figure 1:**
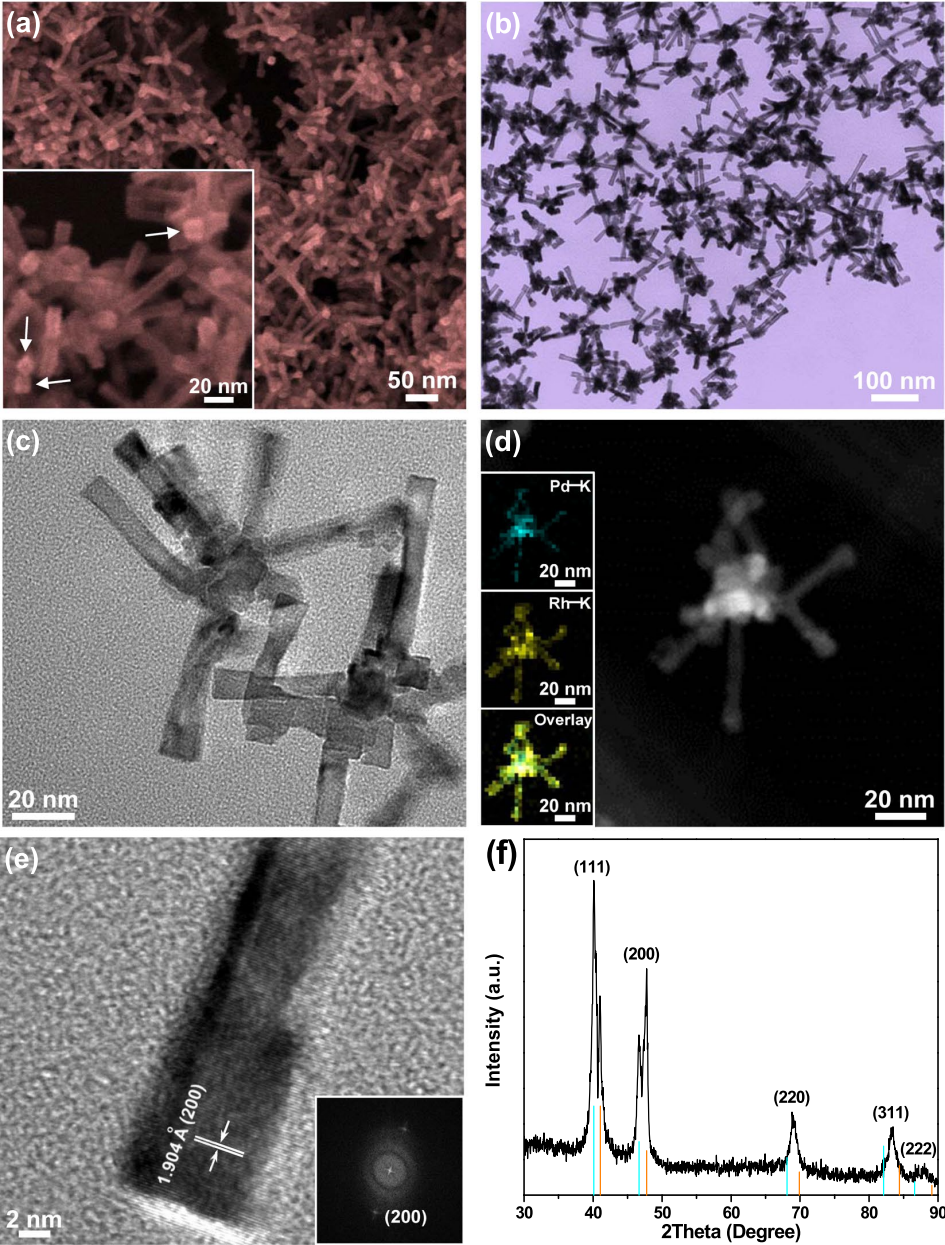
Morphological and structural features of the b-Pd@Rh-NCs. (**a**) Overview SEM, (**b**) low- and (**c**) high-magnification TEM, (**d**) HAADF-STEM, and (**e**) HRTEM images of the b-Pd@Rh-NCs. The insets in panel (**a**), (**d**), and (**e**) show a high-magnification SEM image, the HAADF-STEM-EDS elemental mapping images, and the corresponding FFT image, respectively. The arrows in inset (**a**) indicate the square cross section of branches. (**f**) Powder XRD pattern of the b-Pd@Rh-NCs. The intensities and positions for the pure Pd (cyan, JCPDF no. 05–0681) and Rh (orange, JCPDF no. 05–0685) references are presented as different colorful bars at the bottom according to the JCPDS database.

b-Pd-NCs core was prepared following our previous work and the representative characterization results are presented in Fig. S1 in the Supplementary Materials^40^. Fig. 1a,b show the overview scanning electron microscopy (SEM) and transmission electron microscopy (TEM) images of the b-Pd@Rh-NCs templated by the b-Pd-NCs core, respectively. The rhodium-containing branched nanostructures with branch lengths ranging from 25 to 100 nm are produced in a yield of nearly 100%. Particularly, each branch of the b-Pd@Rh-NCs has a right square prism-like shape. The high-magnification SEM image clearly illustrates that each branch has a square cross section (inset in Fig. 1a). Furthermore, all of the branches have a fairly uniform thickness of 8–13 nm, albeit they have a different length. One can clearly discern that many branches have a core–shell structure based on the different diffraction contrasts arising from two different metal phases with different orientations in the magnified TEM image in Fig. 1c. Note that other branches show no diffraction contrasts because of their core and shell metals with the same orientation. The enlarged TEM image also shows that the surface of each branch is smooth along its entire length. The element distribution revealed by the high-angle annular dark-field (HAADF) scanning TEM (STEM) energy dispersive X-ray spectroscopy (EDS) elemental mapping provides evidence of thin Pd at the inner core and thick Rh at the outside shell for each right square prism-shaped branch (the insets in Fig. 1d). It is clearly visible that each Pd branch with 3–5 nm in diameter is completely encapsulated by a uniform Rh shell with 2–4 nm in thickness along the branch length and at the end of branches (also see Fig. S2), forming a complete core–shell structure. This is because the small difference in the lattice constants (2.3%) between Rh (3.803 Å) and Pd (3.890 Å) facilitates the epitaxial growth of Rh atoms on the surface of Pd branches.

The chemical composition of b-Pd@Rh-NCs is determined from EDS quantitative evaluation (Fig. S3), which gives a bulk Rh: Pd atomic ratio of 65.2:34.8, in good accord with the value (62.3:37.7) obtained from inductively coupled plasma atomic emission spectrometry (ICP-AES) analysis. The high resolution TEM (HRTEM) image in Fig. 1e shows the lattice spacing of 1.904 Å, which perfectly matches the interplanar distance of the Rh (200) plane, confirming the shell composition of Rh. The assignments of the corresponding fast Fourier transform (FFT) pattern reveal that the long-axis for all Rh shells is exclusively parallel to the 〈100〉 direction. On the basis of HRTEM imaging, the right square prism shape of all the branches suggests that they are enclosed exclusively by four Rh {100} facets. Compared to the X-ray diffraction (XRD) diffractogram of the b-Pd-NCs (Fig. S1b), two sets of XRD peaks from the outer fcc Rh shell and inner b-Pd-NCs core are observed for the b-Pd@Rh-NCs, revealing a two-phase core@shell nanostructure rather than a bimetallic nanoalloy (Fig. 1f). In particular, the XRD diffractogram from the Rh shell shows that the relative intensity of the (200) diffraction peak is stronger than that of the (111) peak. Such a {200}-enhanced diffraction pattern implies the (100) textured nanocrystal pattern, further confirming the branch shell with selectively exposed {100} facets. As suggested by other researchers^24,41^, the special formation of Rh {100} facets can be attributed to the synergistic stabilization and etching effects of PVP and Br^−^ ions through their chemisorption on Rh {100} facets as well as the appropriate growth kinetics (i.e., reduction rate of Rh ions).

**Figure 2:**
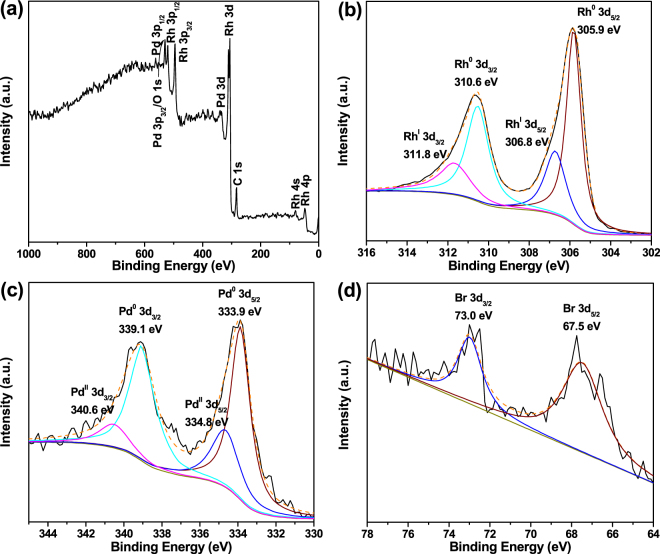
XPS spectra collected from the b-Pd@Rh-NCs. (**a**) XPS survey spectrum, (**b**) Rh 3d, (**c**) Pd 3d, and (**d**) Br 3d detail spectra.

X-ray photoelectron spectroscopy (XPS) is employed to analyze the surface chemical compositions and chemical states of b-Pd@Rh-NCs. Besides the adventitious C species, the survey spectrum in Fig. 2a shows the characteristic peaks of constituent Rh and Pd elements along with weak O signal. The O peak originates from the surface oxides associated with Rh(I)–O. Figure 2b,c reveal one doublet for the Rh 3d and Pd 3d region, respectively, which arises from the spin-orbit coupling of 3d_3/2_ and 3d_5/2_. The strong Rh 3d signals originate from the dominant surface Rh species and can be fitted into two pairs of doublets by deconvolution (Fig. 2b), one pair of strong peaks centered at 305.9 (3d_5/2_) and 310.6 eV (3d_3/2_) and the other of weak peaks at 306.8 (3d_5/2_) and 311.8 eV (3d_3/2_), which can be attributed to the metallic Rh(0) and the adsorbed or re-oxidized ionic Rh(I) species, respectively^42,43^. The very weak Pd signals observed in the survey spectrum and in the Pd 3d region in Fig. 2c reveal its low abundance at the surface, clearly indicating that the majority of the b-Pd-NCs cores are located more than 2 nm below the surface of the b-Pd@Rh-NCs. Similarly, the Pd 3d signals are deconvoluted into two pairs of doublets, which can be assigned to the dominant metallic Pd(0) and minor ionic Pd(II) species that are adsorbed or reoxidized at the Rh/Pd interface during the growth of Rh shell (Fig. 2c). The Rh/Pd atomic ratio is measured to be 12.4:1 at the surface. The weak peaks in the Br 3d region can be attributed to the residual Br^−^ ions binding to the Rh(100) surface of branches (Fig. 2d).

The amount of the precursor, rhodium(II) acetate dimer ([(Rh(Ac)_2_]_2_), plays a key role in controlling the morphology of branched Pd@Rh (b-Pd@Rh) NCs. Reducing the amount of [(Rh(Ac)_2_]_2_ in the reaction system to 5.0 mg leads to insufficient growth of Rh {100} faces, i.e., the formation of the b-Pd@Rh NCs with ill-defined surface structures and thinner arms (4–8 nm in diameter) (Fig. S4a). When the amount of [(Rh(Ac)_2_]_2_ increases to 8.5 mg, each obtained b-Pd@Rh NC has a larger central core while the size and surface structure of its branches nearly remain unchanged in comparison with the b-Pd@Rh-NCs discussed above (Fig. S4b). Evidently, the excess Rh precursor brings about a further growth of Rh at the central core. The b-Pd-NCs cores provide the preformed nucleation sites and thus promote a homogeneous nucleation and growth rate for the Rh shell on them. As a result, compared to the Rh nanocubes reported previously^24,25,27^, the right square prism shaped arms of the b-Pd@Rh-NCs have extended {100} facets in a shape yield of 100%, thus affording us pure Rh {100} facets for investigating the crystal face-dependent electrocatalysis.

### Electrocatalytic activity of b-Pd@Rh-NCs for HEO

**Figure 3:**
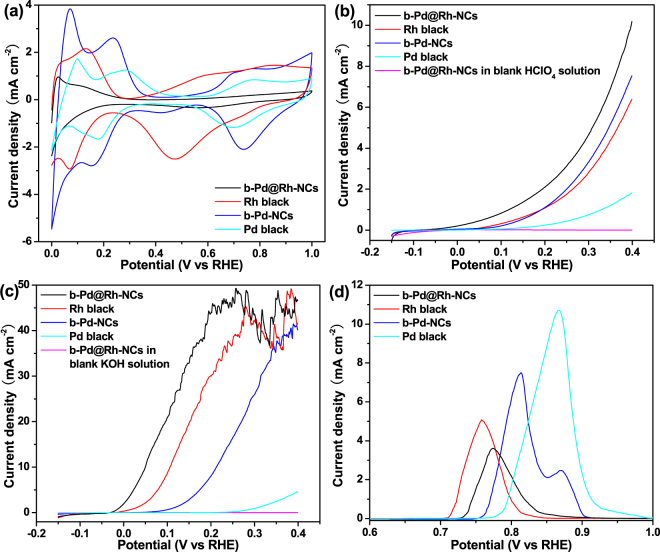
The HEO activities of the b-Pd@Rh-NCs, Rh black, b-Pd-NCs, and Pd black. (**a**) CV recorded in Ar-purged blank 0.1 M HClO_4_ at a scan rate of 50 mV s^−1^. (**b** and **c**) LSV curves obtained in Ar-saturated (**b**) 0.1 M HClO_4_ and (**c**) 1 M KOH aqueous solutions containing 0.10 M hydrazine. The scan rates for all LSV curves are set at 2 mV s^−1^. (**d**) CO stripping voltammetry of various catalysts in 0.1 M HClO_4_ solution at a scan rate of 20 mV s^−1^. The current has been derived by subtracting the first cycle from the second cycle (see Fig. S6).

For electrocatalysis measurements, various catalysts were directly deposited on a glass carbon (GC) working electrode instead of loading them on carbon black support in order to reduce the capacitance current from the carbon support. The adsorbed capping agents on the b-Pd@Rh-NCs sample was completely removed by thoroughly washing it with hot water (75 °C) and ethanol (50 °C) alternatively three times again under vigorous sonication before preparing the catalytic electrode, as evidenced by the Fourier transform infrared (FT-IR) spectrum in Fig. S5. Figure 3a displays the cyclic voltammograms (CVs) of the b-Pd@Rh-NCs, commercial Rh black (Aladdin), b-Pd-NCs, and commercial Pd black (Sigma-Aldrich), which are recorded in argon-purged 0.1 M HClO_4_ for comparison. The characteristic hydrogen desorption/adsorption region (0‒0.35 V_RHE_) and the oxygenated species adsorption region (above 0.43, 0.35, 0.48, and 0.58 V_RHE_ for the b-Pd@Rh-NCs, Rh black, b-Pd-NCs, and Pd black, respectively) can be clearly identified for these electrocatalysts. Apparently, our results suggest that the Rh (100) terraces on the b-Pd@Rh-NCs are more resistant to electrochemical oxidative adsorption than the Rh black. This is consistent with a previous study that demonstrated that Rh surfaces with large terraces are oxidized more difficultly than stepped surfaces^44^. Accordingly, the upper potential limit for the HEO on the b-Pd@Rh-NCs catalyst is restrained to 0.40 V_RHE_ to prevent the catalyst from surface oxidation and the consequent surface reconstruction (i.e., the deterioration of the Rh {100} faces) according to its CV curve. For consistent comparison, the upper potential limit for other references is also set at 0.40 V_RHE_, while their separate onset potential of surface oxidation is different.

Figure 3b compares the linear sweep voltammetry (LSV) curves for the HEO obtained on the different electrocatalysts using argon-purged 0.1 M HClO_4_ as the electrolyte. Among various catalysts, the largest anodic current of the HEO reaction is obtained on the b-Pd@Rh-NCs during positive scan in the range of measured potentials. Concomitantly, the background current of the b-Pd@Rh-NCs electrode is very low (<0.01 mA cm^−2^) in blank supporting electrolyte, which verifies its highest catalytic activity towards the HEO. It can be also observed that the onset potential of the HEO increase in the sequence b-Pd@Rh-NCs < Rh black ≈ b-Pd-NCs < Pd black (see Table 1). Furthermore, the onset potential (−0.098 V_RHE_) on the b-Pd@Rh-NCs is much more negative than that (*ca*. 0.05 V_RHE_) on bulk Rh(100) single crystal reported by Álvarez-Ruiz *et al*.^15^. When using alkaline solution as the supporting electrolyte, the onset potential increases in the order of b-Pd@Rh-NCs < Rh black < b-Pd-NCs ≪ Pd black (see Table 1). Among various electrocatalysts, Pd black exhibits the lowest specific and mass activities in acidic and alkaline solutions (Table 1), which can be attributed to the stronger adsorption of reaction intermediates on its surface presumably due to its surface structure. Pd black will not be discussed in the latter section and all the catalysts mentioned thereafter refer to other three samples. Note that the catalytic currents are more than one order of magnitude higher in alkaline electrolyte than those obtained in acidic electrolyte (Fig. 3c). For each catalyst, the anodic current increases until it reaches a plateau because the well-known diffusion-limited oxidation of N_2_H_4_ dominates. The current plateau occurs at *ca*. 0.20, 0.28, and 0.37 V_RHE_ for the b-Pd@Rh-NCs, Rh black, and b-Pd-NCs, respectively. This phenomenon is largely caused by diffusion-limited Faradaic reaction when the rate of hydrazine consumption at the potentials more positive than the above values is higher than the rate of diffusion of hydrazine onto the catalytic electrode. In addition, the ultimate limiting current densities obtained by the different catalysts are comparable (45 ± 5 mA cm^−2^). Note that compared to the HEO in the HClO_4_ solution, the continuous release of a larger amount of gas bubbles on the surface of each catalyst electrode leads to the drastic fluctuation of currents in the limiting current plateau region in 1 M KOH solution.

**Table 1:** The parameters for comparing the electrocatalytic performance of various catalysts towards the HEO.

Sample (0.2 mg cm^−2^)	Onset potential(V *vs* RHE)		Specific activity^a^ (mA cm^−2^)		Mass activity^a^ (mA mg_NM_^−1^)		${{\bf{Q}}}_{{{\bf{H}}{\boldsymbol{/}}{\bf{Cl}}{\bf{O}}}_{{\bf{4}}}^{{\boldsymbol{-}}}}$(mC cm^−2^)	Q_CO_ (mC cm^−2^)	ECSA (m^2^ g^−1^)
	in HClO_4_	in KOH	in HClO_4_	in KOH	in HClO_4_	in KOH			
b-Pd@Rh-NCs	−0.098	−0.032	0.266	5.557	10.49	219.37	2.42	11.59	20.14
Rh black	−0.063	−0.018	0.117	3.258	5.39	150.24	6.05	13.54	23.53
b-Pd-NCs	−0.062	−0.01	0.107	0.775	5.53	40.18	11.24	22.21	26.44
Pd black	−0.053	0.163	0.015	0.001	1.23	0.12	5.09	84.64	42.32

^a^The specific and NM-based mass activities are calculated at the applied potential of 0.2 V *vs* RHE.

The adsorption (desorption) charge in the hydrogen/perchlorate (H/ClO_4_^−^) region (0‒0.35 V_RHE_) is first used to compare the electrochemically active surface area (ECSA) of catalysts (see Fig. 3a), although it is difficult to obtain the real ECSA values due to the lack of literature values corresponding to the relation between the charge and a H/ClO_4_^−^ monolayer adsorption for these electrocatalysts. The charge of H/ClO_4_^−^ adsorption/desorption (Q_H/ClO4_^−^) for each catalyst is obtained by the deduction of the double-layer region on its CV curve and summarized in Table 1. Apparently, based on the Q value, the ECSA of the b-Pd@Rh-NCs is smaller than that of the Rh black benchmark and most likely also smaller than that of the b-Pd-NCs despite the presence of hydrogen absorption into Pd. To precisely compare the ECSA and probe the surface properties of various catalysts, CO stripping curves are shown in Fig. 3d and S6, in which a dominant CO stripping peak at around 0.80 V_RHE_ is observed for each catalyst. The calculated charges (Q_CO_) for CO oxidation on different catalysts are summarized in Table 1. The ECSAs of the b-Pd-NCs and Pd black are derived from the equation ECSA = (Q_CO_/0.42)/*M*_c_, where the oxidation charge of a CO monolayer on Pd surface is assumed to be 0.42 mC cm^−2^ and *M*_c_ is the catalyst loading on a GC electrode, and calculated to be 26.44 and 42.32 m^2^ g^−1^, respectively. In the case of Rh-based catalysts, the ECSAs can be evaluated based on the normalized Q_CO_ value of 0.2877 mC cm^−2^ for the oxidation charge of a CO monolayer on Rh^45^. The ECSAs of the b-Pd@Rh-NCs and Rh black are derived from the equation ECSA = (Q_CO_/0.2877)/*M*_c_ and consequently calculated to be 20.14 and 23.53 m^2^ g^−1^, respectively.

Compared to the Rh black benchmark, the smaller ECSA but higher activity of the b-Pd@Rh-NCs suggests that the Rh {100} faces is intrinsically more active towards the HEO. Recently, Álvarez-Ruiz and co-workers argued that among the low-index surfaces of Rh, Rh (100) surface is most active for the HEO based on the specific electronic and geometrical configurations for hydrazine dehydrogenation^15^. Correspondingly, they assumed that the most favorable configuration of Rh-NH-NH-Rh adsorbate is formed on the Rh (100) terraces with a minimum strain in the Rh d_γ_ normal orbit‒N bond^15^. This optimal N_2_H_4_ adsorption array on the Rh surface may be applied into the HEO catalyzed by the b-Pd@Rh-NCs. In addition, for multiple-component NM nanocatalysts, a variety of mechanisms, such as electronic effects^34^, lattice strains^35^, and synergistic effects^46^, have been proposed to explain the enhanced catalytic performance. In our case, a coupled electronic effect between Rh shell and the underlying Pd core via hybridization of Rh d states and the neighboring Pd d states could also contribute to the enhancement of electrocatalytic activity of the b-Pd@Rh-NCs (see Fig. S7 and the related discussion). Furthermore, the onset potential and peak potential of CO oxidation on the b-Pd@Rh-NCs are higher than those on Rh black, indicating less steps and defects on the surface of the b-Pd@Rh-NCs. This conclusion is consistent with a previous report that the activity of Rh electrodes towards CO oxidation increases with step density and/or number of defects^44^.

Evidently, in our case, the catalytic HEO reaction is easier and kinetically more rapid in alkaline electrolyte than in acid electrolyte. This can be attributed to the facile formation of the determinant OH_ad_ species on the catalyst surface through oxidative discharge of OH^−^ ions (M + OH^−^ ↔ M‒OH_ad_ + e^−^) during positive scan in alkaline solution, which facilitates the conversion of the H_ad_ species, produced from the dissociative adsorption of hydrazine (N_2_H_4_ → 4H_ad_ + N_2_), into water (H_ad_ + OH_ad_ → H_2_O). Thus, the overall HEO reaction (N_2_H_4_ + 4OH^‒^ → 4H_2_O + N_2_ + 4e^−^) is accelerated. It is assumed that among these catalysts, the b-Pd@Rh-NCs probably provides the best balance between OH_ad_ and H_ad_ coverage as well as the most favorable surface atomic configuration of Rh(100) lattice for the HEO. To interpret the mechanism of the HEO reaction on the most active b-Pd@Rh-NCs electrocatalyst, the Tafel slopes estimated from potential versus log *j* plot are shown in Fig. S8a. In acidic electrolyte, the Tafel slopes are 53 and 289 mV dec^−1^ in the low and high potential regions, respectively, indicating that the mechanism possibly follows the processes of (i) b-Pd@Rh-NCs + NH_2_NH_3_^+^  ↔ b-Pd@Rh‒NHNH_2_ + 2 H^+^  + e^−^ and then (ii) b-Pd@Rh‒NHNH_2_ → b-Pd@Rh-NCs + N_2_ + 3 H^+^  + 3e^−^, where the rate-determining step is believed to be the first dehydrogenation step (i) based on the comparison of the current and previous literature data^12,13^. However, in alkaline solution, much lower Tafel slopes of 15 and 206 mV dec^−1^ are separately obtained in the low and high potential regions (Fig. S8b). This result suggests that the rate-determining step is the step (ii) in the range of low potentials and then shifts to the step (i) with increasing potential^15,16^. In addition, the dramatically enhanced Tafel slopes with potential both in acidic and alkaline electrolytes can be unambiguously ascribed to the diffusion-controlled kinetics occurrence (i.e., mass-transport limitation at higher potentials) for the HEO.

**Figure 4:**
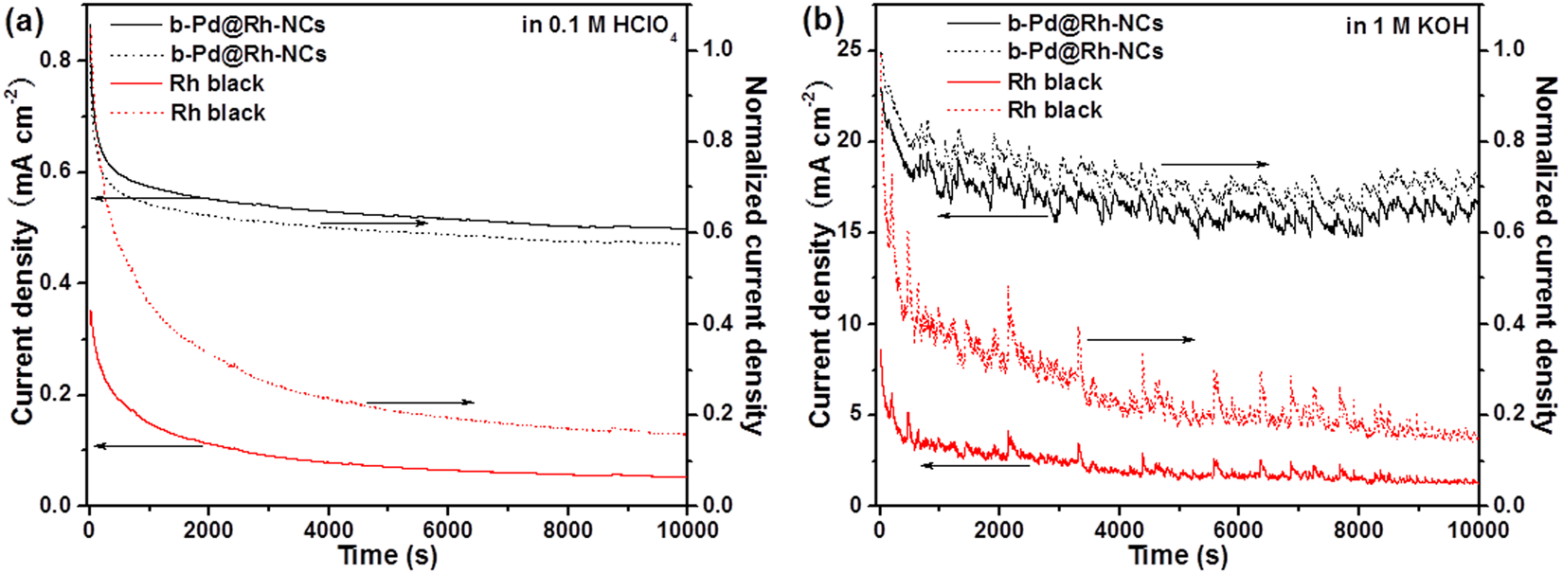
Chronoamperometry (CA) and the corresponding normalized CA curves of the b-Pd@Rh-NCs and Rh black electrocatalysts for the HEO in an Ar-purged solution of (**a**) 0.10 M hydrazine and 0.1 M HClO_4_ and (**b**) 0.10 M hydrazine and 1 M KOH. The CA measurements are conducted at an applied potential of 0.1 V_RHE_.

The long-term stabilities of the b-Pd@Rh-NCs and Rh black reference for the HEO are evaluated by chronoamperometry (CA) measurements, as shown in Fig. 4. For each catalyst, a significant decay in the initial period observed in acidic or alkaline solution is due to a strong adsorption of the intermediate species from the HEO onto the catalyst surface, giving rise to the poisoning of the catalyst surface. Note that the electrocatalytic activities of the b-Pd@Rh-NCs and Rh black catalysts reflected from the currents in their CA profiles obtained in acidic and alkaline solutions are close to those derived from the LSV measurements in Fig. 3b,c. The drastic fluctuation of CA curves recorded in 1 M KOH solution can be attributed to the release of large amount of gas bubbles from the electrode surface, modifying the mass transport of hydrazine hydrate molecules. For the b-Pd@Rh-NCs, the catalytic current decays to 58% or 71% of its initial value at the end of measurements in acidic or alkaline solution, respectively, which is much higher than the corresponding value (16% or 15%) obtained from the Rh black. The b-Pd@Rh-NCs catalyst has a higher stability for the HEO against poison because of its exposed (100) surface. Evidently, the Rh (100) terraces of the b-Pd@Rh-NCs exhibit a considerably weaker affinity to the poisonous intermediate species than the Rh black surface with rich surface defects, steps, and kinks, thereby resulting in a much higher activity maintenance. In addition, the b-Pd@Rh-NCs catalyst has a high structural stability against aggregation due to its branched features, as evidenced by no visible variations in the morphology of the b-Pd@Rh-NCs catalyst (see Fig. S9).

**Figure 5:**
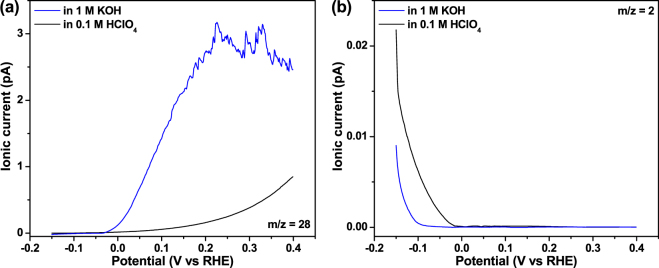
Ionic mass currents for (**a**) nitrogen (m/z = 28) and (**b**) hydrogen (m/z = 2) recorded during DEMS measurements of LSV obtained on the b-Pd@Rh-NCs surface in 0.1 M HClO_4_ or 1 M KOH aqueous solution containing 0.10 M hydrazine.

The products of the HEO reaction on Rh surface have not been studied previously while such a study is important for determining and improving fuel efficiency. We further monitor the evolution of the gaseous products from the HEO on the b-Pd@Rh-NCs using an *in-situ* DEMS technique. Figure 5 illustrates the ionic mass currents of N_2_ at *m*/*z* = 28 and H_2_ at *m*/*z* = 2 as a function of potential obtained by DEMS measurements of LSV in the presence of 0.10 M hydrazine in 0.1 M HClO_4_ or 1 M KOH. It is found that the N_2_ mass response versus potential behaves in a manner similar to the corresponding LSV curves when scanning from −0.1 to 0.4 V_RHE_. The N_2_ ion currents gradually increase with potential in both solutions, as shown in Fig. 5a. The pronounced H_2_ ionic mass currents from the electrochemical reduction of water and possible nonfaradaic hydrazine decomposition are clearly visible at potentials more negative than ca. −0.05 and −0.02 V_RHE_ in 1 M KOH and 0.1 M HClO_4_, respectively, and rapidly decrease with increasing potential (see Fig. 5b). Eventually, no molecular hydrogen at potentials more positive than aforementioned values can be observed, nor was the formation of ammonia found during the entire potential sweeping process (data not shown for brevity). Notably, H_2_ ionic currents are absent within the potential ranges of −0.05 – 0 and −0.02 – 0 V_RHE_ in alkaline and acidic electrolytes, respectively, implying that the parallel chemical hydrazine decomposition (N_2_H_4_ → N_2_ + 2H_2_) is negligible at least within the above potential ranges. This is because molecular H_2_ would be unambiguously detectable if the nonfaradaic hydrazine decomposition took place considering the fact that the negative potentials versus RHE disable the electro-oxidation of the evolved H_2_. Compared to a previous study demonstrating that the rate of the chemical hydrazine decomposition may be elevated with increasing electrode potential^6^, in our case, the reaction rate of the parallel chemical decomposition relative to that of the HEO decreases to a great extant because no H_2_ ionic currents are detected near 0 V_RHE_. Therefore, this is beneficial for improving the fuel efficiency of direct hydrazine fuel cells with the b-Pd@Rh-NCs as an anode electrocatalyst.

## Conclusions

In summary, the b-Pd@Rh-NCs with preferential exposure of Rh {100} facets have been successfully fabricated by growing Rh shell on the pre-synthesized b-Pd-NCs core using PVP in combination with Br^−^ ions as a capping regent. A comparative analysis of voltammetric data reveals that the b-Pd@Rh-NCs electrocatalyst shows significantly enhanced electrocatalytic performance for the HEO in both acidic and alkaline electrolytes due to its favorable surface atomic configuration, unique nanostructure, and possible coupled electronic effect. All the catalysts including the b-Pd@Rh-NCs, Rh black, and b-Pd-NCs show much higher activities towards the HEO in alkaline than in acidic solution. The Tafel slopes demonstrate the different mechanisms of the HEO on the b-Pd@Rh-NCs catalyst in acidic and alkaline solutions. Concomitantly, the HEO on the b-Pd@Rh-NCs surface is highly selective towards N_2_ and H_2_O and greatly eliminates the unwanted nonfaradaic chemical decomposition pathways based on on-line DEMS analysis, leading to a maximum energy efficiency of the b-Pd@Rh-NCs-based hydrazine fuel cell. Therefore, our studies pave the way for developing novel branched metallic nanostructures with well-defined crystalline faces for electrocatalytic applications.

## Methods

### Synthesis of materials

All chemicals were purchased from various commercial sources and used without further purification. Water used for all experiments was high purity water (Millipore water, 18 MΩ).

*Synthesis of b-Pd-NCs core*. A reaction solution was prepared by mixing 40.0 mL of 0.40 M hexadecyltrimethylammonium bromide (CTAB, >99%, Sigma), 20.0 mL of 5.0 mM CuBr_2_ (99%, Alfa Aesar), and 20.0 mL of 0.20 M L-ascorbic acid (L-AA, >99%, Alfa Aesar) in a 200 mL three-neck round-bottom flask, and then the solution was heated in a 70 °C water bath for 1 h under vigorous magnetic stirring. Next, 20.0 mL of 1.0 mM H_2_PdCl_4_ (Sinopharm Chemical Reagent Co., Ltd. (SCRL)) were added to the aforementioned solution, and then the ultimate reaction mixture was kept at 70 °C for 2 h under vigorous magnetic stirring, followed by centrifugation and washing with hot water (~45 °C) three times for purifying the precipitates. Eventually, the resulting product was dispersed in 4 mL of ethylene glycol (EG, 99%, SCRL) and used as the core template in the next step of synthesizing b-Pd@Rh-NCs.

*Synthesis of b-Pd@Rh-NCs*. Typically, 110.0 mg of L-AA, 110.0 mg of KBr (99%, SCRL), 260.0 mg of poly(vinyl pyrrolidone) (PVP, *M*_W_ = 40 000, SCRL), 18.0 mL of EG, and b-Pd-NCs in 4 mL of EG were mixed together in a 100 mL three-neck round-bottom flask and preheated at 110 °C for 2 h under vigorous magnetic stirring. Afterwards, the mixture was heated to 180 °C by a heating mantle, followed by adding 7.0 mL of EG solution containing 7.0 mg of [(Rh(Ac)_2_]_2_ (>98%, Alfa Aesar) dropwise at a rate of 3.5 mL min^–1^ while stirring vigorously. After completing the addition of the [(Rh(Ac)_2_]_2_ solution, the reaction was allowed to proceed for 20 min at 180 °C until the resulting solution turned brown. Finally, the as-prepared b-Pd@Rh-NCs sample was collected by centrifugation and then washed with hot water (45 °C) and ethanol (45 °C) alternatively three times for later characterization.

To probe the effects of the amount of [(Rh(Ac)_2_]_2_ on the morphology of b-Pd@Rh NCs, 5.0 or 8.5 mg of [(Rh(Ac)_2_]_2_ was also used for growing Rh shell on the b-Pd-NCs under otherwise the same conditions.

### Electrochemical measurements

Electrochemical measurements were carried out in a three-electrode configuration consisting of a b-Pd@Rh-NCs, Rh black, b-Pd-NCs, or Pd black working electrode, a platinum wire counter electrode, and an Ag/AgCl/KCl (3 M) reference electrode in single compartment. The working electrodes were prepared by pipetting an aqueous suspension of each preceding electrocatalyst onto a glassy carbon (GC) electrode (Pine Research Instrumentation, 5 mm in diameter, 0.196 cm^2^) to obtain a catalyst loading of 0.2 mg cm^−2^, followed by casting a diluted Nafion (Aldrich) solution (0.05 wt%). The GC electrode was consecutively polished using 1 and 0.05 μm alumina and then thoroughly washed with water and dried before preparing working electrodes. The electrocatalytic HEO were performed in a 0.1 M HClO_4_ or 1 M KOH solution containing 0.1 M hydrazine hydrate using a CHI 660D electrochemical analyzer (CH Instruments, Chenhua Co., Shanghai, China) at ambient temperature (~20 °C). All electrolyte solutions were purged with pure argon gas for at least 30 min to remove air prior to each experiment. All potentials were quoted versus the reversible hydrogen electrode (V_RHE_). CO stripping measurements were performed in 0.1 M HClO_4_ by using Ag/AgCl (3 M KCl) as the reference electrode. After the potential was held at 0.1 V_RHE_ for 30 min while CO was purged into the solution, the glassy-carbon electrode was removed from the solution and immersed in a fresh 0.1 M HClO_4_ which was pretreated by purging with Ar gas for 30 min. LSV curves were recorded by scanning from 0 to 1.2 V_RHE_ at a scan rate of 10 mV s^−1^.

### DEMS measurements

The on-line DEMS measurements were carried out using a Pfeiffer Vacuum ThermoStar GSD 301 T3 benchtop mass spectrometer with a setup consisting of two differentially pumping chambers. The connection between the mass spectrometer and the cell was achieved through a steel capillary connected to a glass tube where a polyetheretherketon holder was attached. This tip inlet was placed inside the electrolyte of the electrochemical cell at a 10–20 μm distance from the catalyst electrode surface using a positioning system to allow the diffusion of gaseous species into the differentially pumped area of the spectrometer. The HEO products were monitored at the mass/charge ratios of *m*/*z* = 28 (N_2_) and *m*/*z* = 2 (H_2_).

## Electronic supplementary material

Supplementary Information 
