# Supplementary material for: Branched Pd@Rh core@shell nanocrystals with exposed Rh {100} facets: an effective electrocatalyst for hydrazine electro-oxidation
Source: Sci Rep. 2017 Nov 28;7:16465. doi: 10.1038/s41598-017-16776-6 (PMC5705708; doi:10.1038/s41598-017-16776-6)
Supplement: Supplementary file 1 — Supplementary Information [file 41598_2017_16776_MOESM1_ESM.pdf]

Supplementary materials

Branched Pd@Rh core@shell nanocrystals with exposed Rh {100} facets: an effective electrocatalyst for hydrazine electro-oxidation

Guojing Wang, Shengchang Jing, and Yiwei Tan*

State Key Laboratory of Materials-Oriented Chemical Engineering, School of Chemistry and Chemical Engineering, Nanjing Tech University, Nanjing 210009, China, Email: ytan@njtech.edu.cn, Tel: +86-25-83172267

Experimental section

Characterization of materials

Scanning electron microscopy (SEM) micrographs were obtained using a Hitachi S-4800 field-emission scanning electron microscope operating at 5 kV. Transmission electron microscopy (TEM) and high resolution TEM (HRTEM) micrographs were taken using a FEI Tecnai G2 F20 S-Twin transmission electron microscope operating at an accelerating voltage of 200 kV. Scanning TEM (STEM) images and energy-dispersive X-ray spectroscopy (EDS) elemental maps were acquired by the high-angle annular dark field (HAADF) mode using the same transmission electron microscope. Specimens for TEM, including HRTEM and STEM, investigations were prepared by depositing a drop of diluted nanoparticle dispersion in water onto 300 mesh carbon-coated molybdenum grids. X-ray photoelectron spectroscopy (XPS) measurements were performed using a PHI5000 VersaProbe (ULVAC-PHI) spectrometer with an energy analyzer, employing a monochromatized microfocused Al-Kα (*hv* = 1486.58 eV) X-ray source. Samples for XPS measurements were pretreated by repeated cycles of Ar+ ion sputtering to obtain clean surfaces of the samples. The binding energy (BE) of core level was calibrated by setting the adventitious C 1s peak at 284.8 eV. Survey spectrum of each sample in the binding energy range of 0–1000 eV and core level spectra of elemental signals were recorded at a resolution of 1 and 0.125 eV, respectively. The X-ray diffraction (XRD) patterns were recorded using a Rigaku Smartlab diffractometer with Cu Kα radiation (λ = 1.5406 Å) operating at 40 kV and 100 mA at a scanning rate of 0.06 ^{o}sec^{-1}. Quantitative elemental analysis of the chemical composition was determined by EDS and inductively coupled plasma atomic emission spectrometry (ICP-AES, Prodigy, Leeman LABs Inc., USA, λ = 165–800 nm, As = 200 nm) measurements. Sample for ICP-AES measurements was dissolved in aqua regia. Fourier transform infrared spectra (FT-IR) were recorded in a transmission mode in the range of 400–4000 cm^{-1} on a Nicolet iS 50 FT-IR Spectrometer (Thermo Scientific) using KBr pellets.

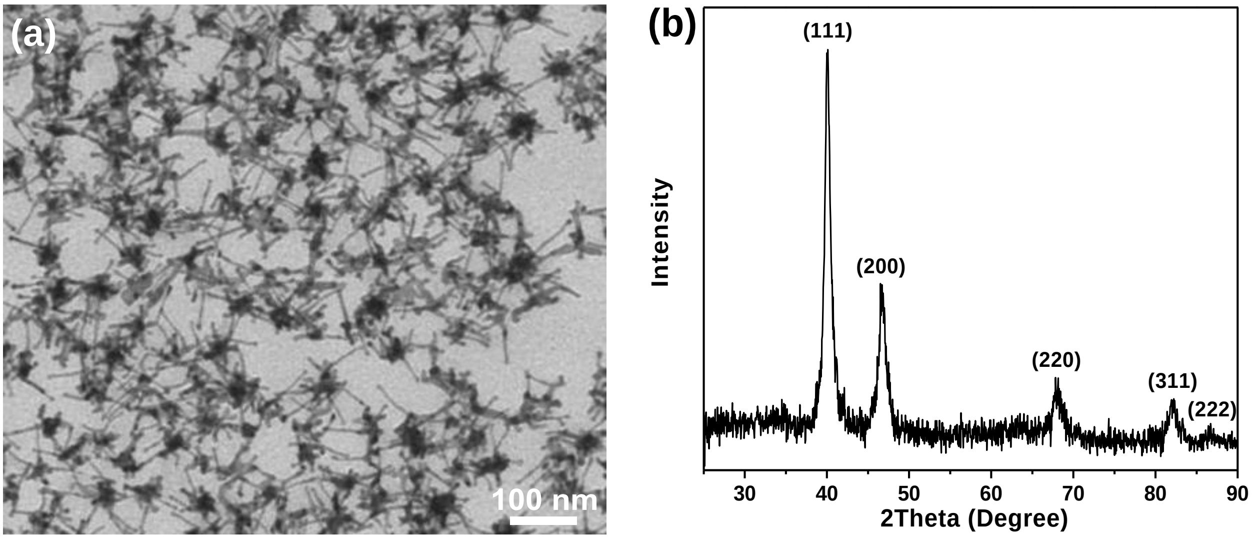

Figure S1. (a) TEM image and (b) XRD pattern of the pre-synthesized b-Pd-NCs.

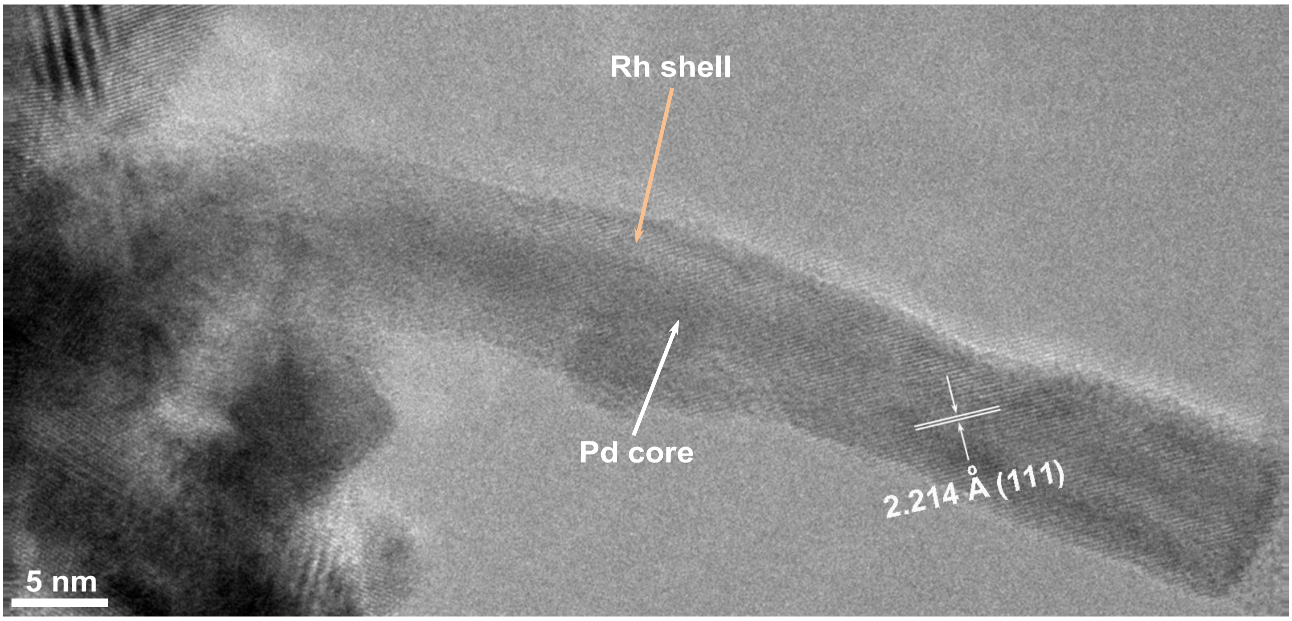

Figure S2. TEM image of the b-Pd@Rh-NCs showing the Pd core and Rh shell.

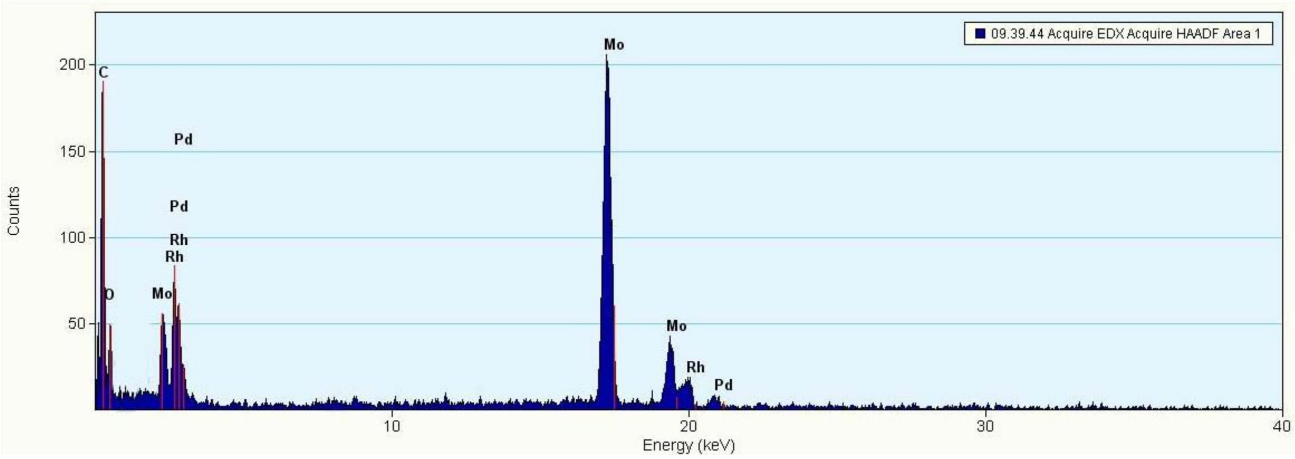

Figure S3. EDX spectrum recorded from the b-Pd@Rh-NCs on a molybdenum grid.

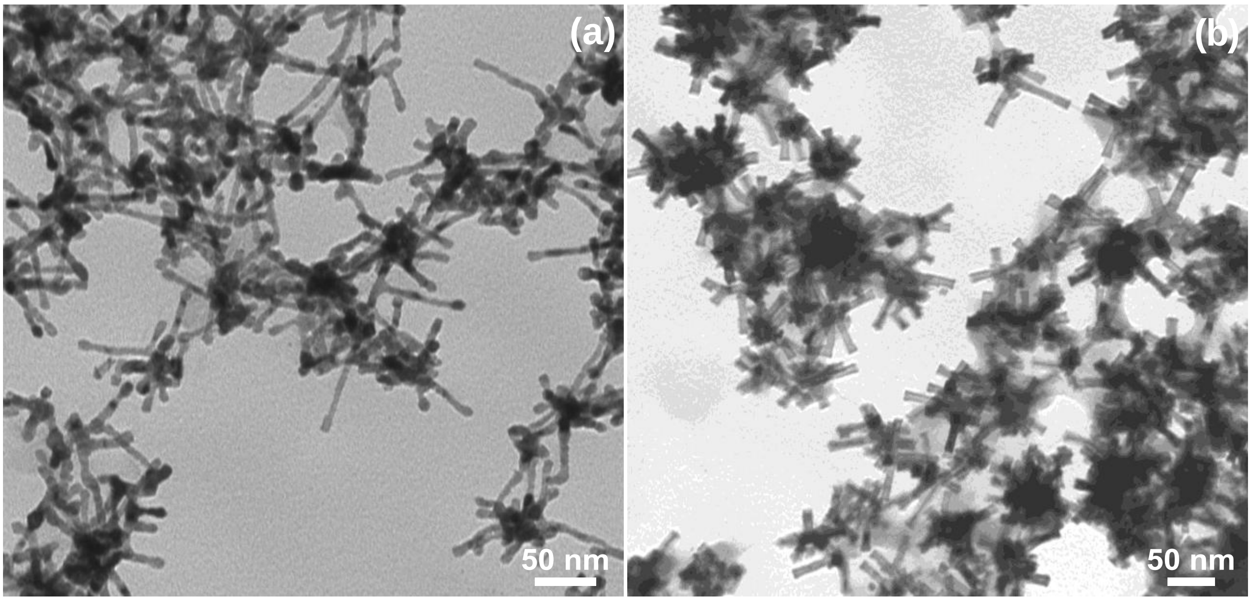

Figure S4. TEM images of the branched Pd@Rh NCs synthesized by using (a) 5.0 mg and (b) 8.5 mg of rhodium(II) acetate dimer in the reaction systems.

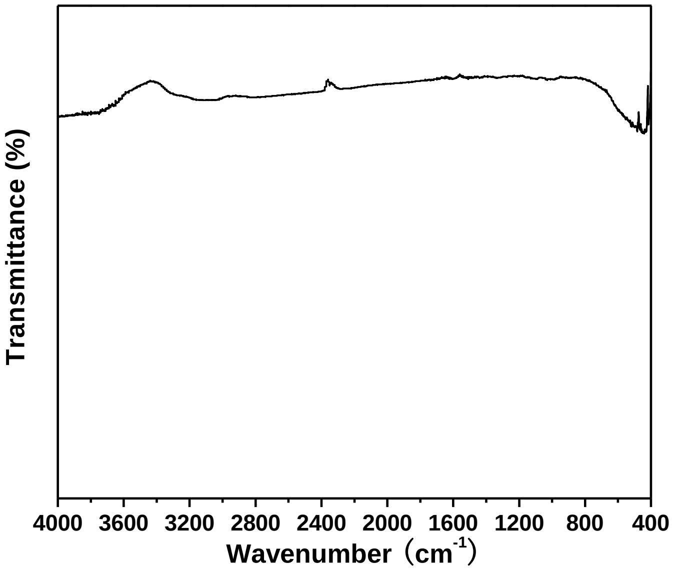

Figure S5. FT-IR spectrum of the b-Pd@Rh-NCs sample used for preparing catalytic electrodes for the HEO. No absorption bands originating from organic functional groups can be observed, indicating that various capping agents have been removed from the b-Pd@Rh-NCs sample surface after thoroughly washing.

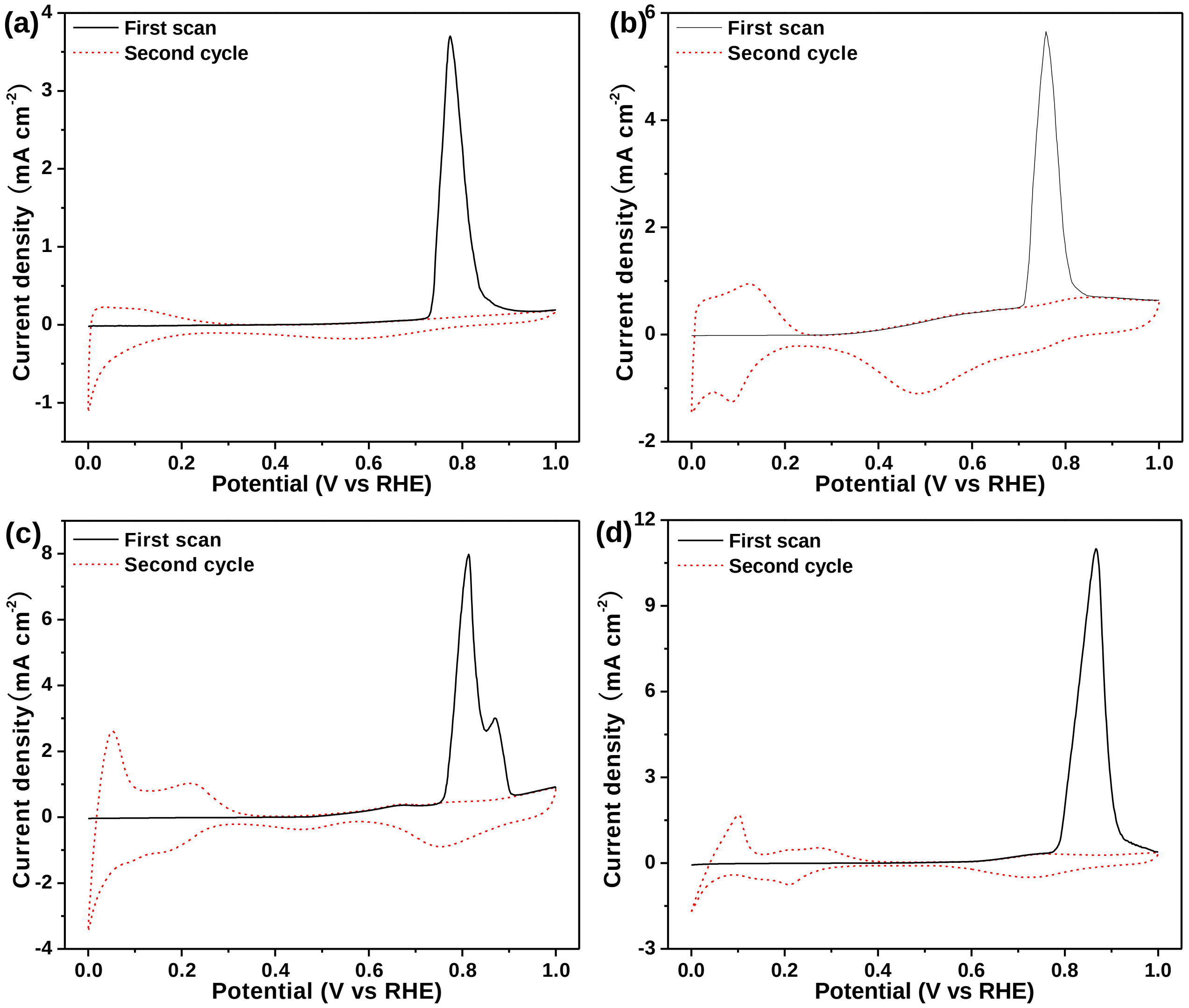

Figure S6. CO stripping voltammetry of the (a) b-Pd@Rh-NCs, (b) Rh black, (c) b-Pd-NCs, and (d) Pd black in 0.1 M $HClO_4$ solution at a scan rate of 20 mV s^{-1}.

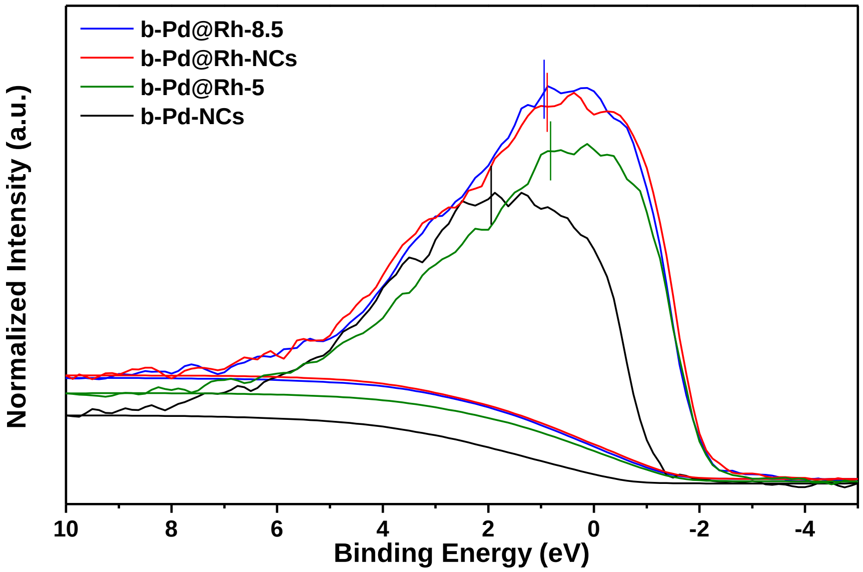

Figure S7. Valence band spectra collected from the b-Pd-NCs and different b-Pd@Rh NCs. The vertical dash bars indicate the d-band center of each catalyst. The b-Pd@Rh-5, b-Pd@Rh-NCs, and b-Pd@Rh-8.5 denote the samples prepared using 5.0, 7.0, and 8.5 mg of rhodium(II) acetate dimer, respectively.

Figure S7 compares the valence band (VB) spectra of the b-Pd-NCs and different b-Pd@Rh NCs to examine the modification of the surface electronic structure. The d-band center position of each catalyst is determined from the weighted average energy of its VB spectrum through subtraction of the Shirley background and then integration. For consistent comparison of the integration results, an upper integration limit of 10 eV position is chosen for each VB spectrum that is subtracted from the corresponding Shirley background. The d-band centroid values of the b-Pd@Rh-5, b-Pd@Rh-NCs, b-Pd@Rh-8.5, and b-Pd-NCs are 0.82, 0.88, 0.90, and 1.91 eV, respectively. Apparently, the b-Pd@Rh NCs exhibits the much lower d-band centroid values than the b-Pd-NCs. The very small difference of the d-band centroid values between b-Pd@Rh-5 and b-Pd@Rh-NCs implies that increasing the Rh shell thickness does not significantly affect the surface electronic structure of the b-Pd@Rh NCs. At the same time, the b-Pd@Rh-NCs and b-Pd@Rh-8.5 samples with the similar Rh shell thicknesses show negligible changes in the d-band centroid value. In some previous reports, the authors proposed that charge transfer from one constituent (e.g., Pd) to another (e.g., Rh) is responsible for the changes in the d band center position. However, in our case, the atomic electronegativity of Pd (2.20) does not differ much from that of Rh (2.28). In addition, it is difficult to explain the charge compensation of the positive charges left in the donor (Pd) domain after electron transfer. Therefore, electron transfer is not expected to occur between Pd and Rh. We present a more reasonable explanation on the substantial downshift in d-band energy centre of various b-Pd@Rh NCs. The decrease in d-band centroid value of each b-Pd@Rh NCs sample is correlated with the formation of new electronic states above the Fermi level due to the hybridization of Rh d states and the neighboring Pd d states. Thus, the d-band energy centre shifts to a lower BE in order to preserve the number of states below and above the Fermi level. The formation of pure Rh shell suggests that there are no effects of mutual coordination of core-shell components on the surface electronic structure. In addition, the small lattice mismatch between Pd and Rh and the relatively thick Rh shell also exclude the effects of lattice strain on the catalytic activity of the b-Pd@Rh-NCs.

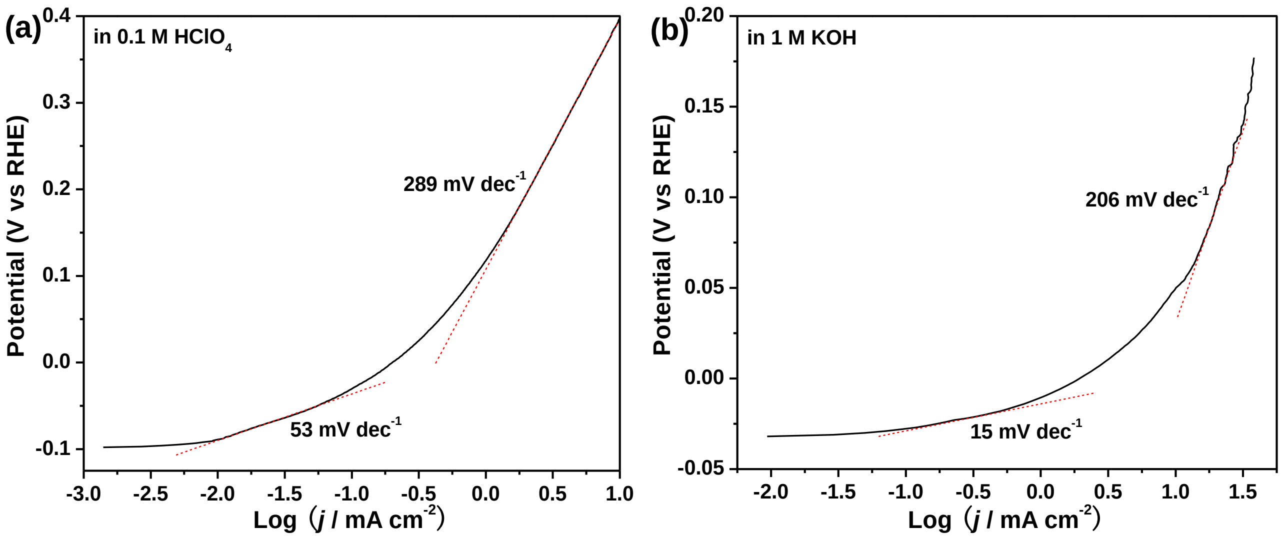

Figure S8. Tafel slope analysis of the polarization curves for the HEO on the b-Pd@Rh-NCs catalyst in (a) 0.1 M $HClO_4$ and (b) 1 M KOH solution containing 0.10 M hydrazine.

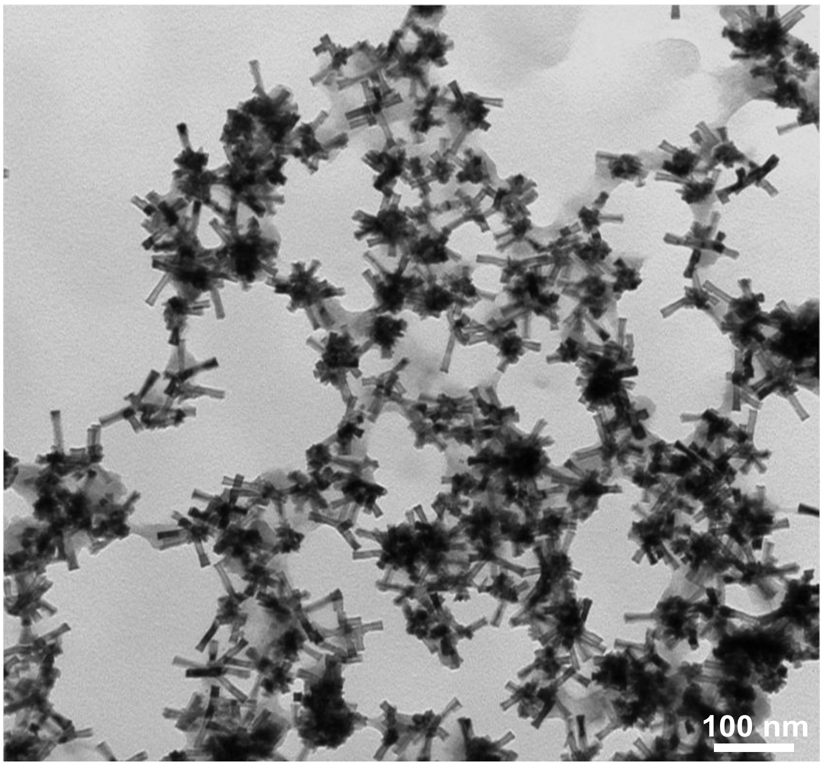

Figure S9. TEM image of the b-Pd@Rh-NCs catalyst obtained after the 10000 s-CA stability test. The specimen for TEM observation is prepared by sonicating the catalyst electrode in water and then by dropcasting the water suspension onto a carbon-coated copper grid.
